# Multi‐modal direct LINAC parameter prediction for pancreatic VMAT: An optimization‐free approach

**DOI:** 10.1002/mp.70656

**Published:** 2026-08-30

**Authors:** Zixu Guan, Yukine Shimizu, Takahiro Iwai, Michio Yoshimura, Takashi Mizowaki, Mitsuhiro Nakamura

**Affiliations:** ^1^ Department of Advanced Medical Physics Graduate School of Medicine Kyoto University Kyoto Japan; ^2^ Department of Radiation Oncology and Image‐Applied Therapy Graduate School of Medicine Kyoto University Kyoto Japan

**Keywords:** auto‐planning, deep learning, VMAT

## Abstract

**Background:**

Pancreatic cancer Volumetric Modulated Arc Therapy (VMAT) planning presents a significant dosimetric challenge due to the high‐dose gradients required to spare adjacent, radiosensitive organs at risk (OARs) like the stomach and duodenum. This anatomical complexity has limited the scope of automated planning for this site. Broadly, while deep learning (DL) has been introduced to streamline treatment planning, most existing models only predict intermediate outputs, such as dose distributions or fluence maps, which still necessitate a subsequent, computationally expensive inverse optimization step on a treatment planning system.

**Purpose:**

To address these challenges, we aim to develop an optimization‐free fully automated VMAT planning framework for pancreatic cancer. As a key component of this system, this study introduces a DL model designed to directly generate machine parameters from dose distributions and anatomical contours.

**Methods:**

A total of 200 vmat plans for pancreatic cancer (prescription: 42 Gy in 15 fractions) were retrospectively collected. The dataset was randomly split into training (*n *= 170), validation (*n* = 10), and testing (*n* = 20) sets. The proposed Multi‐modal, Attention & Transformer‐Enhanced U‐Net (MATE‐UNet) utilizes beam's‐eye‐view (BEV) projections of the reference dose distribution and anatomical contours to directly predict machine‐executable multi‐leaf collimator (MLC) apertures and Monitor Units (MUs). The proposed model was benchmarked against baseline U‐Nets (using contour‐only, dose‐only, and combined inputs) on the testing set. Model accuracy was assessed using the Dice Similarity Coefficient (DSC) for MLC and Mean Absolute Error (MAE) for MU, while plan quality was evaluated using clinical dose‐volume histogram (DVH) metrics and Conformity Index (CI), Homogeneity Index (HI), and Gradient Index (GI).

**Results:**

MATE‐UNet achieved a clinical acceptance rate of 100% (20/20), compared to 70% for the best‐performing baseline model. In terms of prediction accuracy, the proposed model achieved a DSC of 0.9553 ± 0.0042 for MLC apertures and an MAE of 1.960 ± 0.396 for MU. Dosimetric evaluation demonstrated that, relative to the reference plans, MATE‐UNet maintained comparable target coverage, CI (0.773), and HI (0.100), while achieving a significantly improved GI (3.732, *p* < 0.05). Furthermore, MATE‐UNet significantly reduced the V_39Gy_ of the stomach and duodenum, as well as the global maximum dose, compared with the baseline models (*p* < 0.05).

**Conclusions:**

This study demonstrates the feasibility of MATE‐UNet for the direct prediction of VMAT machine parameters without iterative optimization. By leveraging multi‐modal BEV inputs and a Transformer‐enhanced architecture, the proposed framework represents a valuable step toward bridging the gap between dose distributions and clinically usable treatment plans. Although an additional normalization step is required to determine the absolute MUs, MATE‐UNet has the potential to serve as a downstream component of a future fully automated anatomy‐to‐plan pipeline.

## 1 INTRODUCTION

Pancreatic cancer remains one of the most fatal malignancies with a dismal prognosis.^1^ Although surgical resection is the only potential curative treatment, the majority of patients present with locally advanced or metastatic disease at diagnosis, rendering them ineligible for surgery.^2^ Therefore, radiotherapy or neoadjuvant chemoradiotherapy is considered an important treatment modality, especially for patients with locally advanced pancreatic cancer (LAPC) and borderline resectable pancreatic cancer (BRPC).^3^, ^4^, ^5^

Effective pancreatic radiotherapy is challenging due to the proximity of radiosensitive organs at risk (OARs), such as the stomach and duodenum. This proximity creates a dosimetric conflict between achieving high dose coverage for the target and sparing these OARs. Volumetric modulated arc therapy (VMAT) is an advanced technique that enables the delivery of highly conformal dose distributions, minimizing normal tissue toxicity.^6^, ^7^ The VMAT planning process relies on complex inverse optimization, which requires a time‐consuming, trial‐and‐error approach by the human planner. Additionally, this manual intervention introduces variability in plan quality between different planners.^8^, ^9^, ^10^ Automated treatment planning is therefore essential to relieve medical physicists from this repetitive, labor‐intensive and ensure consistent, high‐quality treatment plans.^11^

Current automated planning solutions generally fall into two categories. Knowledge‐based treatment planning (KBP) has been extensively researched, and several commercial systems, such as RapidPlan (Varian Medical Systems, Palo Alto, CA, USA), are now in clinical use.^12^, ^13^ Alongside these KBP methods, deep learning (DL) models have emerged as a powerful alternative, enabling automated planning by accurately predicting intermediate outputs, such as dose distributions or fluence maps.^14^, ^15^, ^16^

However, both KBP and DL‐based approaches share a critical limitation: neither eliminates the final, computationally intensive optimization step, which remains the primary bottleneck to a fully automated planning workflow. KBP models inherently incorporate optimization in their generation of planning parameters.^12^, ^17^ Similarly, the representations predicted by DL models are not directly deliverable and still require subsequent optimization to derive LINAC parameters, including multi‐leaf collimator (MLC) apertures and monitor units (MU).

To address these limitations, an emerging research direction focuses on the direct prediction of LINAC parameters. However, this field remains nascent and existing studies face challenges. For example, Ni et al. predicted MLC apertures but did not address MU prediction, resulting in incomplete LINAC parameters.^18^ Another study utilized the predicted MLC and MU as initial parameters for a subsequent post‐optimization step, rather than for direct delivery.^19^ To the best of our knowledge, the only study that attempted to directly generate fully deliverable LINAC parameters without subsequent optimization reported clinically significant degradation in PTV coverage, failing to consistently satisfy clinical requirements.^20^ Consequently, there is a critical need for an approach that can directly and accurately generate comprehensive LINAC parameters (both MLC and MU) to yield clinically acceptable plans without any further optimization. Importantly, most prior studies have primarily focused on treatment sites such as prostate and breast cancer.^16^, ^18^, ^19^, ^20^, ^21^ In contrast, pancreatic radiotherapy involves substantial dosimetric trade‐offs because the target is located in close proximity to multiple dose‐limiting gastrointestinal OARs (e.g., stomach and duodenum). Consequently, achieving adequate target coverage while simultaneously satisfying stringent OAR constraints requires careful optimization to balance tumor control and toxicity reduction. We hypothesize that an architecture capable of successfully navigating the steep dose gradients and complex OAR‐target trade‐offs inherent in pancreatic treatments demonstrates a level of robustness and generalizability that potentially could scale to less demanding treatment sites. Validating the proposed framework on such a challenging site provides a rigorous proof‐of‐concept, suggesting that direct‐parameter prediction is feasible even in highly demanding anatomical configurations.

In this study, we introduce a novel DL framework, the Multi‐modal, Attention & Transformer‐Enhanced U‐Net (MATE‐UNet), designed to directly generate LINAC parameters for pancreatic cancer VMAT. Specifically, MATE‐UNet converts a patient's multi‐modal beam's‐eye‐view (BEV) projections (dose and anatomical contours) into both MLC apertures and MU values bypassing the conventional TPS optimization process.

## 2 METHODS

### 2.1 Patients

This study was performed in accordance with the Declaration of Helsinki and was approved by the Medical Ethics Committee of the Graduate School of Medicine and Faculty of Medicine, Kyoto University and conducted with permission from the head of the research institution (R1446‐3).

We retrospectively collected 200 VMAT treatment plans for pancreatic cancer patients treated at Kyoto University Hospital between 2010 and 2023. For each case, the dataset comprised DICOM files for the RT Plan, CT Image, RT Dose, and RT Structure Set.

### 2.2 Reference treatment planning

Since the plans were originally generated over a period of more than a decade, they were created using various versions of the Eclipse™ Treatment Planning System (TPS) (Varian Medical Systems, Palo Alto, CA, USA). To eliminate inconsistencies arising from these different TPS versions, all plans were re‐optimized and re‐calculated with a single version of the TPS (Eclipse™ 16), serving as the reference plans. The re‐calculation process utilized the original CT simulations and contours, and was performed according to the following standardized protocol.

Involved target volumes were defined as follows: The gross tumor volume (GTV) comprised the primary tumor and metastatic nodes; the clinical target volume (CTV) was defined as the GTV plus a 5 mm margin and the prophylactic area; and the planning target volume (PTV) was formed by adding a 5 mm margin to the CTV. The planning organ at risk volume (PRV) was created to reduce the dose to the gastrointestinal tract, and the PRV comprised the stomach, and duodenum, with 10 mm, and 5 mm margins, respectively.^5^ Additionally, all plans were designed for a Varian TrueBeam linear accelerator equipped with a High‐Definition 120 (HD120) MLC (featuring 32 inner leaf pairs with a 2.5 mm width and 28 outer leaf pairs with a 5 mm width). The technique employed was a single full‐arc VMAT using a 10 MV Flattening Filter Free (FFF) photon beam.

This arc consisted of 180 control points, defined by a clockwise gantry rotation from 181° to 179° in 2° intervals and utilized a fixed 30° collimator rotation. The prescription dose was 42 Gy (15 fractions), defined by two objectives: D_95_ (the dose covering 95% of the structure) = 42 Gy for the PTV‐PRV (PTV excluding PRV), and D_98_ > 36 Gy for the PTV. These recalculated plans were subsequently validated case‐by‐case by a team of experienced medical physicists from Kyoto University Hospital to ensure compliance with the institution's specific clinical requirements (Table 1).

**TABLE 1:** Clinical requirements for VMAT pancreatic cancer at Kyoto University Hospital.

	Metric	Goal
PTV‐PRV	D_95_	=42 Gy
PTV	D_98_	>36 Gy
Body	D_max_	<115%
Stomach	V_42Gy_	<0.5 mL
	V_39Gy_	<1.0 mL
	V_36Gy_	<20.0 mL
Duodenum	V_42Gy_	<0.5 mL
	V_39Gy_	<1.0 mL
	V_36Gy_	<20.0 mL
Spinal cord	D_max_	<36 Gy
Left kidney	V_20Gy_	<30%
Right kidney	V_20Gy_	<30%
Liver	D_mean_	<30 Gy

Abbreviations: D95 = dose covering 95% of the volume; D98 = dose covering 98% of the volume; Dmax = maximum dose; Dmean = mean dose; PTV = planning target volume; PTV‐PRV = PTV excluding the planning organ at risk volume; V20Gy = volume covered by 20 Gy; V36Gy = volume covered by 36 Gy; V39Gy = volume covered by 39 Gy; V42Gy = volume covered by 42 Gy.

### 2.3 Study design

**FIGURE 1:**
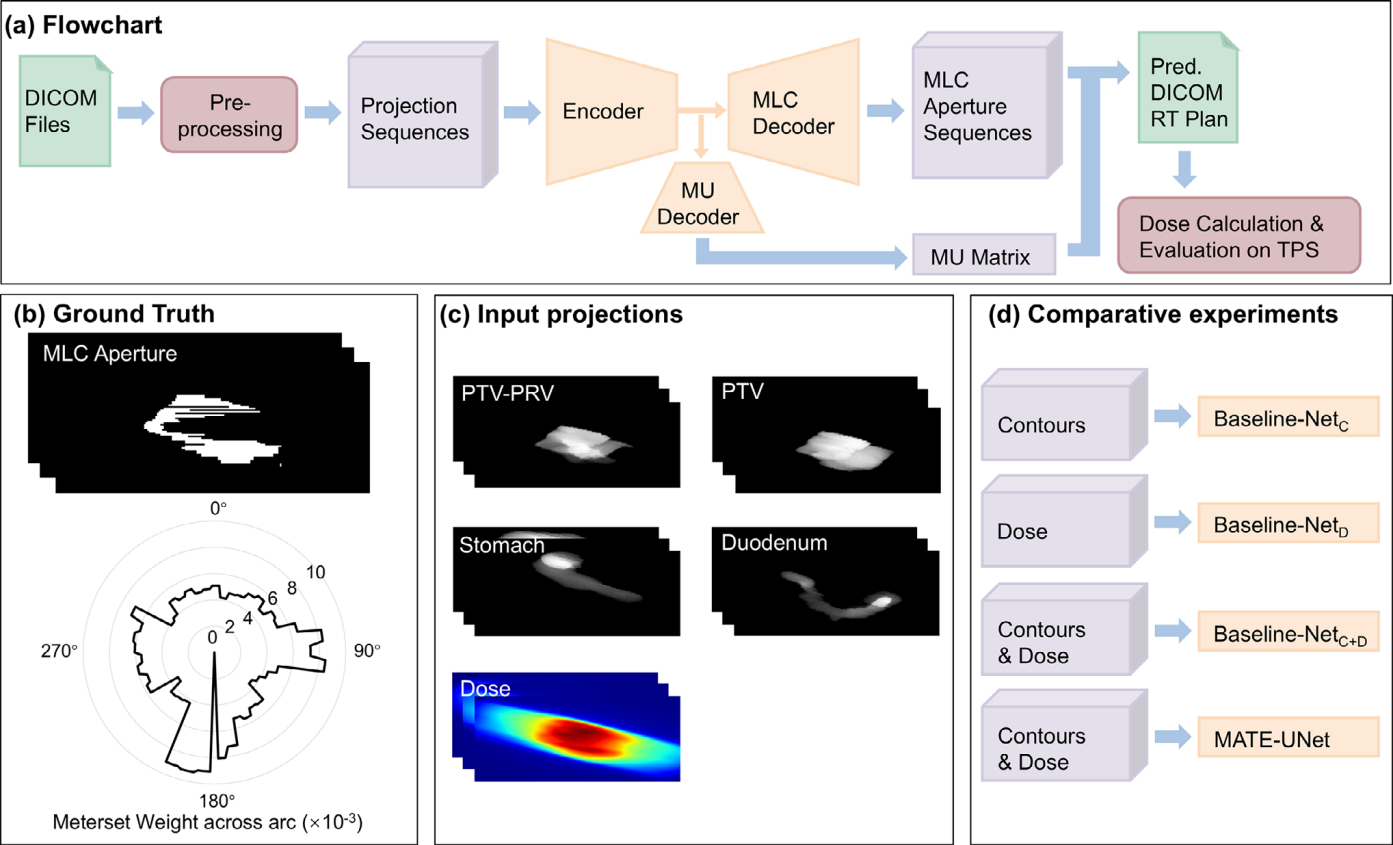
(a) Flowchart of this study, illustrating the transition from DICOM files to deliverable RT plans through preprocessing, deep learning modules, and TPS evaluation. (b) Ground Truth showing MLC aperture sequences and visualization of Meterset Weight across whole arc. (c) Input BEV projection sequences, including PTV‐PRV, PTV, Stomach, Duodenum and Dose. (d) Overview of the experimental design, illustrating the input modality screening and the subsequent evaluation of MATE‐UNet.

The core workflow of our proposed framework is designed to be “optimization‐free”, characterizing a direct inference process for machine parameter generation that eliminates the iterative computational bottleneck inherent in conventional TPS optimization. Figure 1a shows the workflow of this study. Initially, DICOM files of the reference treatment cases were exported from the TPS and preprocessed for DL model training. During this stage, the MLC position matrix from each plan was converted into a sequence of binary mask images, and the corresponding cumulative meterset weights (CMWs) were extracted to represent MU information. Together, these MLC image sequences and meterset weight (MW) matrices served as the ground truth for the DL model (Figure 1b). The model was trained using beam's‐eye‐view (BEV) projections of the dose distribution and anatomical contours as inputs (Figure 1c). The model's predicted outputs—the MLC sequences and MW values—were then postprocessed and compiled into a new DICOM‐RT Plan file, hereafter referred to as the “predicted plan”. Finally, each predicted plan was imported into the TPS for dose calculation, and its quality was evaluated using dose‐volume histogram (DVH) metrics. Additionally, this study investigated four distinct scenarios that used various combinations of inputs and models (Figure 1d). These include three variants of the baseline model: Baseline‐Net_C_ (trained using only contour projections), Baseline‐Net_D_ (trained using only dose projections), and Baseline‐Net_C+D_ (trained using a combination of both contour and dose inputs). Finally, our proposed model, MATE‐UNet, was also evaluated using the combined contour and dose inputs. Throughout this manuscript, the subscripts C and D are explicitly used to denote contour and dose input modalities, respectively.

### 2.4 Data pre‐processing

The DICOM files in our dataset had varying spatial resolutions due to the utilization of two different CT machines (Siemens SOMATOM Definition AS and GE LightSpeed RT16) and differences in acquisition settings. The in‐plane resolution varied depending on patient body size, while the slice thickness differed between scanners (2.0 mm for Siemens and 2.5 mm for GE). As a result, the CT images were acquired at four distinct spatial resolutions: 0.976 × 0.976 × 2.000 mm, 0.976 × 0.976 × 2.500 mm, 1.074 × 1.074 × 2.500 mm, and 1.172 × 1.172 × 2.500 mm. In contrast, the dose files had slice thicknesses of either 2.0 mm or 2.5 mm, with a consistent in‐plane resolution of 2.0 mm.

All data modalities—CT, dose, and structures—were first converted into NumPy arrays. To standardize the data, these arrays were then resampled to a unified resolution of 1.0 × 1.0 × 1.0 mm using trilinear interpolation for dose and CT, and nearest‐neighbor interpolation for structures. Four crucial contours (PTV, PTV‐PRV, stomach, and duodenum) were utilized as the input and each contour was represented as a binary mask, where voxels within a structure were assigned a value of 1 and 0 otherwise. Subsequently, each array was translated so that the treatment isocenter, obtained from the plan file, coincided with the geometric central voxel of the array, employing zero‐padding where necessary to maintain a consistent coordinate frame. Finally, the arrays were cropped to a uniform size of 450 × 450 × 320 voxels, creating a volume of interest (VOI) that fully encompassed the target volumes and the majority of the relevant OARs.

The inputs for the DL model were BEV projection sequences generated from the 3D dose and contour arrays. To simulate the delivery process, projections were generated at 180 control points (CPs) across a full arc (from 181° to 179° clockwise at 2° intervals), incorporating the 30° collimator rotation specified in the reference plans. For the contour arrays, 2D projections ($I_{c}$) were generated via summation along the beam's path. For the dose array, to preserve spatial depth information and approximate the physical attenuation of the photon beam, an attenuation‐compensated forward projection ($I_{d}$) was implemented:

(1) $$I_{d}\;\left(u,v\right)=\sum_{w=0}^{L-1}D\left(u,v,w\right)\cdot \varphi \left(w\right)$$

where w denotes the discrete depth index along the beam axis, progressing from the proximal (beam entry) to the distal (beam exit) boundary within the BEV coordinate system, L represents the total number of sampling layers integrated along this axis, D(u,v,w) is the dose array at the BEV coordinates, and φ(w) is a linear depth‐weighting factor increasing linearly from 0 (at the beam entry) to 1 (at the beam exit) along the beam axis. This process assigned greater weight to distal voxels, effectively preventing the loss of spatial features during the 3D‐to‐2D transformation. The resulting 2D images were stacked into a sequence with dimensions of 180 × 320 × 450 (angular × longitudinal × lateral), where the sequence order strictly followed the temporal delivery of the control points.

The ground truth for the model consisted of two components: MLC sequences and MU information, which were derived from the 200 reference plans. For each control point, the MLC apertures were extracted from the DICOM RT Plan files and converted into 2D binary masks, corresponding to a field size of 220 × 300 mm with a resolution of 1.0 × 1.0 mm. In these masks, a value of 1 represented an open beam path, while 0 indicated a blocked state. The 180 individual masks for each plan were then concatenated to create the initial MLC sequence with dimensions of 180 × 220 × 300. The fractional MUs for each treatment segment were derived from the CMW values in the DICOM files, which represent the cumulative MU fraction ranging from 0 to 1. By calculating the difference between consecutive CMW values, we obtained a 1 × 179 matrix representing the MU fraction per segment for each plan.

To ensure dimensional consistency between the inputs and ground truth and to reduce computational load for model training, a final downsampling step was applied to dose and contours projections and MLC sequences. Each array was first cropped to a size of 200 × 192 mm and then resampled to a coarser resolution of 2.5 × 1.0 mm. This process yielded the final dimensions of 180 × 80 × 192 for all arrays used in model training.

### 2.5 Network architecture

Our baseline model, termed Baseline‐Net, was a multi‐task architecture designed to predict both MLC sequences and an MU matrix from input dose and contours projections. This design was driven by the distinct nature of the two tasks: MLC prediction, which resembles an image segmentation task, was handled by a three‐dimensional U‐Net.^22^, ^23^ The MU prediction, being a regression task, was performed by an additional decoder that connected to the shared encoder of the 3D U‐Net. However, the shared U‐Net encoder within the Baseline‐Net had limitations in multi‐modal feature fusion and capturing global context.^24^, ^25^, ^26^, ^27^ To address this, we proposed an enhanced architecture: the Multi‐modal Attention & Transformer‐Enhanced U‐Net (MATE‐UNet). The specific architectures of both models are detailed in the following sections.

#### 2.5.1 Baseline‐net

The 3D U‐Net component of the Baseline‐Net followed a classic encoder‐decoder architecture. It was composed of three downsampling stages that halved the spatial dimensions of the feature maps, and three corresponding upsampling stages that restored them. The output of the MLC decoder was passed through a final Sigmoid activation function, which constrained the predicted voxel values within the range of 0 to 1. The MU decoder branched from the bottleneck of the 3D U‐Net, taking the latent space feature map as its input. This feature map was first processed by an average pooling layer and then flattened into a one‐dimensional feature vector. Subsequently, the vector was passed through a series of three upsampling blocks, each of which consisted of a 1D transposed convolution layer, followed by batch normalization, a Leaky ReLU activation,^28^ and a dropout layer. Finally, a 1D convolution layer with a single kernel projected the feature representation from the last block to generate the final 1 × 179 MU matrix.

#### 2.5.2 MATE‐UNet

**FIGURE 2:**
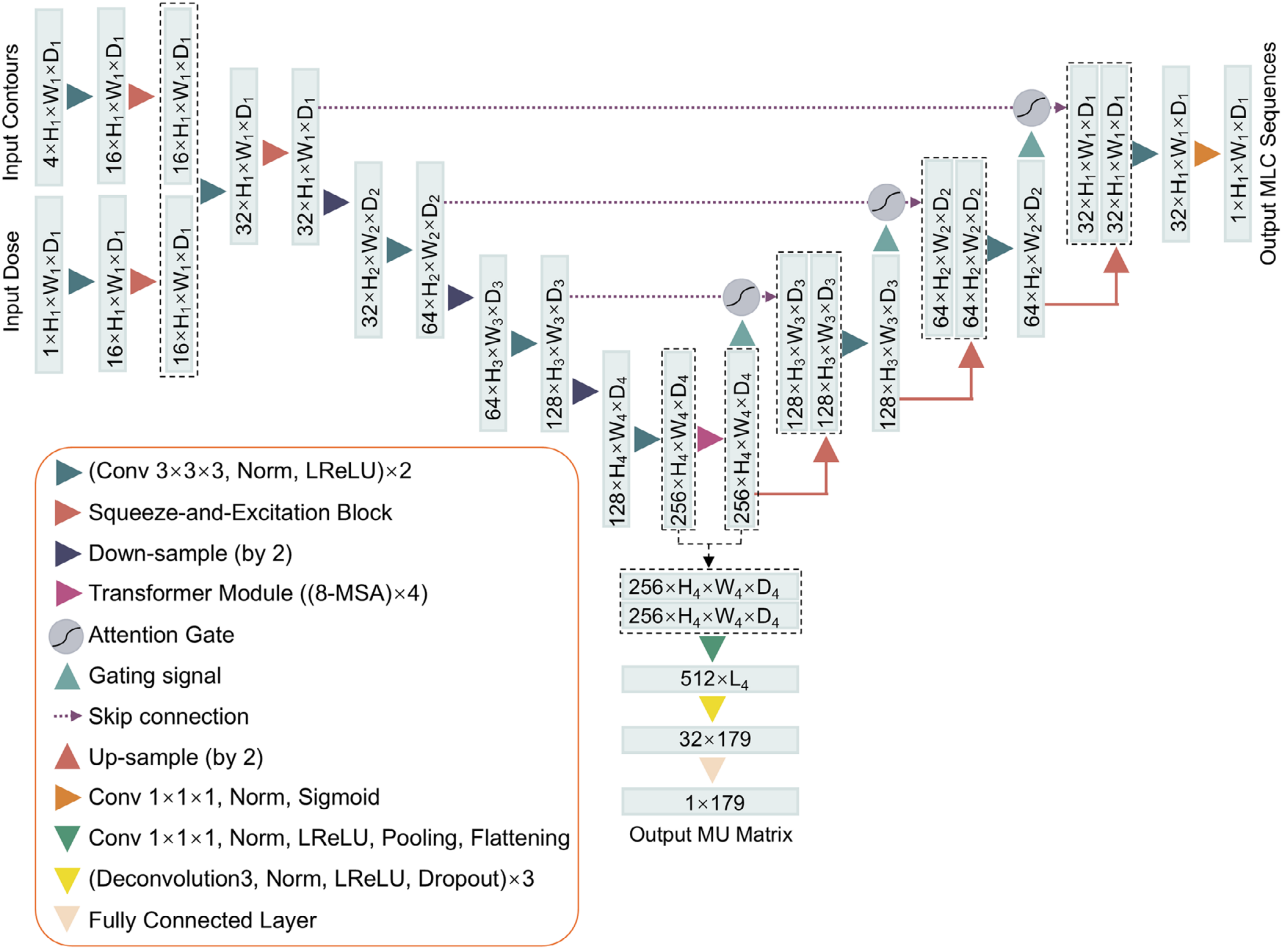
An overview of the proposed MATE‐UNet architecture. The model utilizes a U‐Net encoder‐decoder framework where the encoder progressively downsamples feature maps by a factor of two at each stage. The architecture incorporates three primary enhancements: (a) a multi‐stage feature fusion module processes the initial inputs, (b) AGs are integrated into the skip connections to filter features, and (c) a Transformer module is embedded in the bottleneck to capture global sequential dependencies.

The proposed Multi‐modal Attention & Transformer‐Enhanced U‐Net (MATE‐UNet) (Figure 2) enhanced the Baseline‐Net's encoder‐decoder architecture by integrating three key components. It replaced simple input concatenation with a multi‐stage feature fusion module to handle multi‐modal data. Attention Gates (AGs)^29^ were added to the skip connections to perform spatial filtering, and a Transformer^30^ module was embedded in the bottleneck to capture global sequential dependencies. These components, detailed below, worked jointly to improve the final predictions.

#### 2.5.3 Feature fusion module

The MATE‐UNet took a 5‐channel tensor as input, comprising two distinct modalities: a single‐channel dose projection and four channels of contour projections (PTV, PTV‐PRV, stomach, and duodenum). The feature fusion module was designed to adaptively weigh and recalibrate features both within and between the modalities through the following three stages: first, the dose and contour modalities were processed in parallel branches, where Squeeze‐and‐Excitation (SE)^31^ blocks emphasized important features within each modality. Then, the overall contribution between the modalities was balanced using learnable scalar weights before the branches were fused. Finally, a third SE block was applied to the fused tensor to recalibrate the combined multi‐modal feature space.

#### 2.5.4 Attention gates

Following the fusion module, the 32‐channel tensor was fed into the U‐Net's encoder, which consisted of three downsampling stages similar to the Baseline‐Net. To refine the features passed from the encoder to the decoder, we incorporated AGs into the U‐Net's skip connections. Architecturally, for each skip connection, the gating signal was collected from the coarser scale, while the feature map to be filtered came from the corresponding encoder layer. The attention coefficients computed in the AGs were then multiplied with the input feature map before being concatenated with the upsampled feature map in the decoder.

#### 2.5.5 Transformer module

To capture long‐range sequential dependencies across the arc, we introduced a Transformer module into the U‐Net's bottleneck. The input to the Transformer module was the bottleneck feature map, with learnable positional embeddings. The module itself consisted of 4 encoder layers, each equipped with an 8‐head multi‐head self‐attention mechanism and a GELU activation function.^32^

The output of this Transformer module was then integrated to enhance the MLC and MU decoders. For the MLC decoder, the Transformer's output served two distinct functions: first, it was used directly as the input to the first upsampling block, initiating the decoding path. Second, it was utilized as the gating signal for the AG on the deepest skip connection. On the other hand, the input to the MU decoder combined two feature streams: (1) the original feature representation from the U‐Net's bottleneck, and (2) the global representation from the Transformer. These two tensors were first concatenated along the channel axis. The resulting fused tensor was then passed through a convolutional layer that halved the number of channels, preparing the feature map for the subsequent series of transposed convolution blocks as described previously.

### 2.6 Experiments

The dataset of 200 treatment plans was randomly split into training (*n *= 170), validation (*n *= 10), and testing (*n *= 20) datasets. This allocation was strategically chosen to prioritize the training dataset, ensuring the model could effectively capture the complex anatomical variations and dose‐to‐parameter mappings within a limited total sample size. The validation dataset served exclusively to monitor the training process and guide hyperparameter optimization. Final model performance and generalizability were evaluated solely on the independent testing dataset, which remained entirely unseen during the training and hyperparameter tuning phases. All input data were normalized to the range of 0 to 1 using min‐max scaling. Notably, the ground truth MU matrix was scaled by a factor of 100 prior to training. This scaling was necessary to prevent potential numerical underflow and ensure stable gradient computation, given that the small (∼10^−^^3^) target matrix values.

To simultaneously train the model for both MLC sequence and MU matrix prediction, we employed a multi‐task learning (MTL)^33^ framework that adaptively weighs the loss for each task. The total loss, $L_{total}$, is defined as:

(2) $$L_{total}=\left(e^{-S_{mlc}}\cdot L_{mlc}+S_{mlc}\right)+\left(e^{-S_{mu}}\cdot L_{mu}+S_{mu}\right)$$

where $S_{mlc}$ and $S_{mu}$ are learnable parameters that represent the uncertainty of each task and automatically balance their contributions. $L_{mlc}$ is a weighted sum of Binary Cross‐Entropy (BCE) and Tversky loss^34^ (with empirically determined weights of 0.4 and 0.6, respectively), and $L_{mu}$ is the L1 loss.

The models were optimized using the AdamW^35^ optimizer with differential learning rates: an initial rate of 10^−^^3^ was set for the shared encoder and MLC decoder, and 8 × 10^−^^5^ for the MU decoder. A Cosine Annealing with Warm Restarts schedule was implemented to adjust the learning rate, promoting stable convergence and enhancing model generalization. This approach helped mitigate the risk of overfitting, which was particularly critical given the constraints of the limited dataset. All models were trained for 420 epochs with a batch size of 2. The experiments were conducted on a workstation equipped with an Intel Core i9‐13900KF CPU and a single NVIDIA RTX A6000 GPU (48 GB VRAM), using the PyTorch framework (v2.1.0).

We designed a two‐phase study to systematically optimize both the input configuration and the network architecture.

#### 2.6.1 Phase 1: Input configuration screening

To identify the optimal input configuration, we first conducted a model‐agnostic screening using a Baseline‐Net architecture, including three distinct input configurations: using only contour projections (Baseline‐Net_C_), using only the dose projection (Baseline‐Net_D_), and using a combination of both (Baseline‐Net_C+D_). This phase aimed to determine whether combining contour and dose projections (C + D) provides superior predictive power compared to single‐modality inputs.

#### 2.6.2 Phase 2: Development of MATE‐UNet

Building upon the optimal input configuration identified in Phase 1 (i.e., the C + D combination), we developed the MATE‐UNet to specifically exploit the synergistic features of these dual inputs in Phase 1.

### 2.7 Model evaluation

For the final evaluation on the testing dataset, we selected the model weights from the epoch that achieved the lowest validation loss. The model's prediction accuracy was assessed via the Dice Similarity Coefficient (DSC) for the MLC sequence, which ranges from 0 to 1 (higher is better), and the Mean Absolute Error (MAE) for the MU matrix, where lower values are better.

To assess the clinical applicability of the model's output, a full dosimetric evaluation was performed. The predicted MLC binary masks were converted to HD120 leaf positions, setting the jaw positions to match the aperture height with a 2.0 mm lateral margin. Additionally, the predicted segmental MU matrix was transformed into CMW for each CP. These machine parameters were then used to generate a new DICOM RT Plan file, termed the “predicted plan”. Specifically, an arbitrary total MU (e.g., 100 MU) was temporarily assigned to each predicted plan. These plans were subsequently imported into the TPS Eclipse™, where their 3D dose distribution were calculated using the Acuros XB algorithm (version 16). During this process, the TPS enforced hardware constraints, including leaf speed limits and physical tolerances specific to the LINAC, ensuring that the generated plans are compatible with clinical delivery systems.

Finally, the calculated dose distributions were normalized following the same clinical protocol as the reference plans: The 100% prescribed dose covers 95% of the PTV‐PRV, which served as the primary target volume. Through this TPS normalization process, the final absolute MU was automatically calculated. The dosimetric quality was evaluated using standard DVH‐based metrics for targets and OARs. To assess statistical significance, the Wilcoxon signed‐rank test was employed. Accounting for multiple comparisons, the Holm‐Bonferroni correction was applied. A corrected p‐value was considered statistically significant if it was less than the predefined significance level of *α* = 0.05. Statistical comparisons were structured into two distinct categories:

#### 2.7.1 Inter‐model performance evaluation

To analyze the impact of input configurations and architectural designs, specific DVH metrics were compared across three sets: (1) Baseline‐Net_C+D_ versus Baseline‐Net_C_, (2) Baseline‐Net_C+D_ versus Baseline‐Net_D_, and (3) Baseline‐Net_C+D_ versus MATE‐UNet.

#### 2.7.2 Clinical evaluation against reference plans

A comprehensive evaluation was performed to compare the MATE‐UNet plans against the reference plans. In addition to standard DVH metrics, the plan quality was further quantified using the Conformity Index (CI),^36^ Homogeneity Index (HI),^37^ and Gradient Index (GI),^38^ defined as follows:

(3) $$CI=\frac{TV_{PIV}^{2}}{TV\times PIV}$$

where $TV_{PIV}$ is the target volume wrapped by the prescribed isodose, TV is the total target volume, and PIV is the total volume encompassed by the prescribed dose. The CI value ranges from 0 to 1, where a value closer to 1 indicates superior conformity.

(4) $$HI=\frac{D_{2}-D_{98}}{D_{50}}\;$$

where $D_{2}$, $D_{98}$, and $D_{50}$ represent the dose received by 2%, 98% and 50% of the target volume. An HI value closer to 0 indicates an ideal, perfectly homogeneous dose distribution within the target.

(5) $$GI=\frac{V_{50\%}}{V_{100\%}}$$

where $V_{50\%}$ and $V_{100\%}$ represent the volumes encompassed by the 50% and 100% prescribed isodose. A smaller GI value indicates a steeper dose gradient, representing a more rapid dose fall‐off.

## 3 RESULTS

### 3.1 Inter‐model performance

**FIGURE 3:**
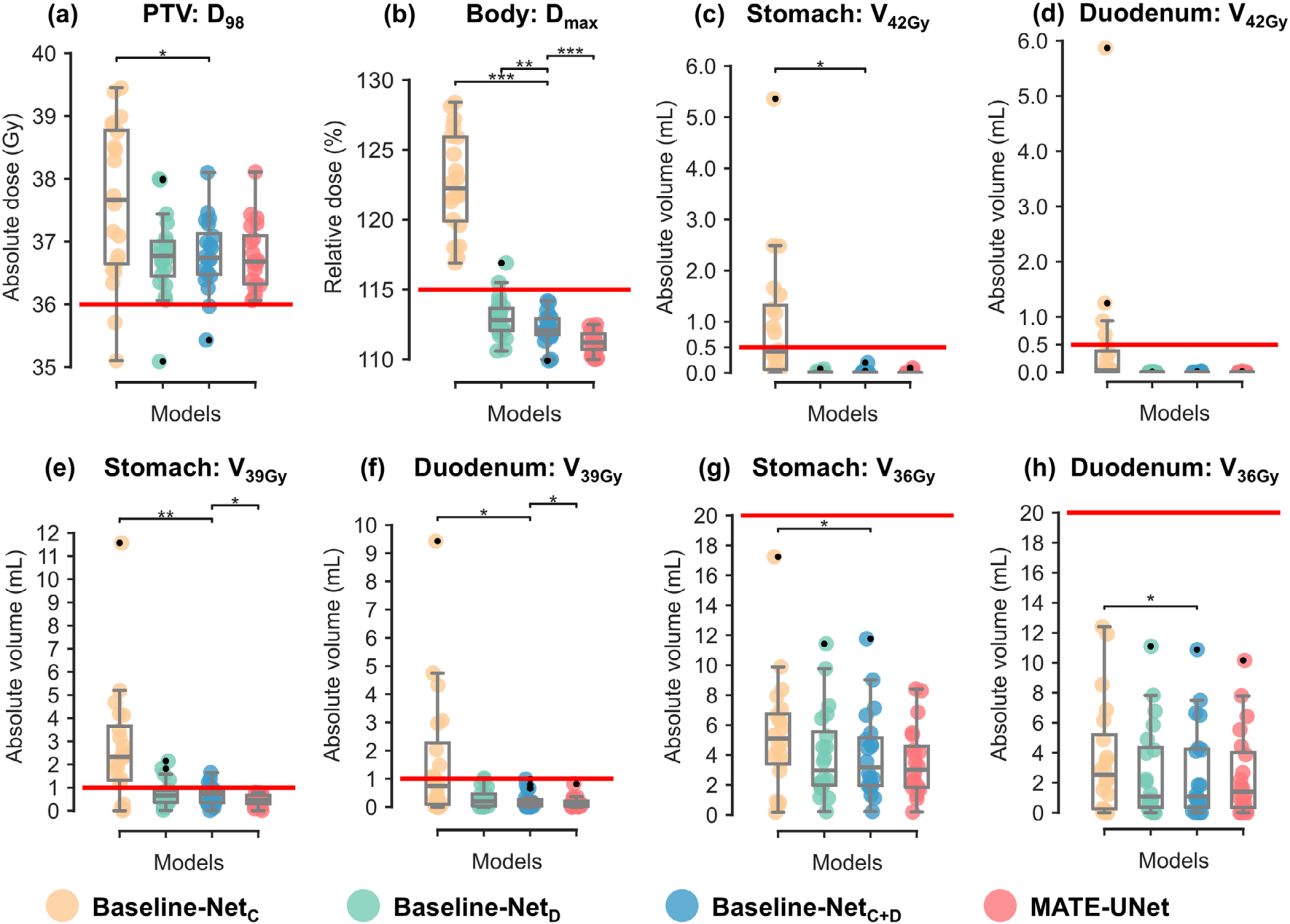
Dosimetric comparison of (a) D98 for PTV, (b) Dmax for body, (c) V42Gy for stomach, (d) V42Gy for duodenum, (e) V39Gy for stomach, (f) V39Gy for duodenum, (g) V36Gy for stomach, (h) V36Gy for duodenum as boxplots. Yellow, green, blue, and red dots symbolize individual data of predicted plans by Baseline‐NetC, Baseline‐NetD, Baseline‐NetC+D, and MATE‐UNet, respectively. The red line represents the clinical requirement. Significant differences between data are marked with a single asterisk (*p* < 0.05), a double asterisk (*p* < 0.01), and a triple asterisk (*p* < 0.001).

Quantitative analysis on the testing dataset (*n* = 20) revealed a clear performance hierarchy determined by input configurations (Table 2). Phase 1 (Input Configuration Screening) indicated that Baseline‐Net_C+D_ achieved the highest accuracy among the baseline models, significantly outperforming (*p* < 0.05) Baseline‐NetC on nearly all metrics (with the exception of V42Gy for duodenum), and showing a significant improvement (*p* < 0.05) over Baseline‐NetD on Dmax for the body as well (Figure 3).

**TABLE 2:** Quantitative comparison on testing dataset (20 plans) for baseline and proposed models.

	DSC for MLC		MAE for MU	
Models	Mean	SD	Mean	SD
Baseline‐Net_C_	0.8487	0.0149	9.36	1.40
Baseline‐Net_D_	0.9482	0.0052	5.87	0.90
Baseline‐Net_C+D_	0.9499	0.0043	5.70	0.92
MATE‐UNet	**0.9553**	**0.0042**	**1.96**	**0.40**

*Note*: Bold highlights the best results of the table. The subscripts C and D denote the contour and dose inputs, respectively.

Abbreviations: DSC = dice similarity coefficient; MAE = mean absolute error; MLC = multi‐leaf collimator; MU = monitor unit; SD = standard deviation.

In Phase 2, comparing the proposed architecture with the optimal baseline, MATE‐UNet demonstrated superior quantitative performance in MAE for MU, achieving a 65.6% reduction relative to Baseline‐Net_C+D_ (Table 2). Specifically, as shown in Figure 3, MATE‐UNet exhibited significant dosimetric improvements (*p* < 0.05) over Baseline‐Net_C+D_ regarding D_max_ for body and V_39Gy_ for stomach and duodenum.

In terms of clinical acceptability, MATE‐UNet attained a 100% (20/20) clinical goal success rate. This performance substantially surpassed the success rates of the baseline models: 70% (14/20) for Baseline‐Net_C+D_ and 65% (13/20) for Baseline‐Net_D_ and 0% (0/20) for Baseline‐Net_C._

### 3.2 Clinical performance against reference plan

A comprehensive dosimetric comparison between the MATE‐UNet generated plans and the reference plans is summarized in Table 3. Both plan sets successfully satisfied all predefined clinical goals for the 20 testing cases.

**TABLE 3:** DVH metrics comparison on testing dataset (20 plans) for MATE‐UNet plans and reference plans.

			Mean (SD)			
	Metric	Goal	MATE‐UNet Plan	Reference Plan	Pred—Ref	*p‐*value
PTV‐PRV	D_95_	=42 Gy	42.00 (0.00)	42.00 (0.00)	0.00	–
PTV	D_98_	>36 Gy	36.76 (0.52)	36.61 (0.50)	0.14	0.122
Body	D_max_	<115%	111.20 (0.79)	109.83 (0.96)	1.38	**0.002**
Stomach	V_42Gy_	<0.5 mL	0.01 (0.02)	0.00 (0.02)	0.01	0.916
	V_39Gy_	<1.0 mL	0.46 (0.24)	0.27 (0.22)	0.18	**0.009**
	V_36Gy_	<20.0 mL	3.56 (2.27)	3.13 (2.13)	0.43	**0.027**
Duodenum	V_42Gy_	<0.5 mL	0.00 (0.00)	0.00 (0.00)	0.00	1.000
	V_39Gy_	<1.0 mL	0.16 (0.19)	0.09 (0.07)	0.08	**0.038**
	V_36Gy_	<20.0 mL	2.55 (2.84)	2.48 (2.82)	0.07	1.000
Spinal cord	D_max_	<36 Gy	22.66 (3.45)	22.43 (3.45)	0.23	0.953
Left kidney	V_20Gy_	<30%	10.79 (5.71)	10.83 (5.45)	−0.04	1.000
Right kidney	V_20Gy_	<30%	3.15 (5.41)	3.00 (5.60)	0.16	1.000
Liver	D_mean_	<30 Gy	5.47 (2.22)	5.46 (2.19)	0.01	1.000

*Note*: Statistically significant differences are marked by bold numbers in the table.

Abbreviations: D95 = dose covering 95% of the volume; D98 = dose covering 98% of the volume; Dmax = maximum dose; Dmean = mean dose; Pred = predicted plan; PTV = planning target volume; PTV‐PRV = PTV excluding the planning organ at risk volume; Ref = reference plan; SD = standard deviation; V20Gy = volume covered by 20 Gy; V36Gy = volume covered by 36 Gy; V39Gy = volume covered by 39 Gy; V42Gy = volume covered by 42 Gy.

Target coverage remained highly consistent between the two groups. Following normalization, the D_95_ for the PTV‐PRV was identical at 42 Gy. For the PTV, the D_98_ achieved by MATE‐UNet (36.76 Gy) was comparable to the reference plans (36.61 Gy), with no statistically significant difference observed (*p* = 0.122). Regarding global dose indicators, MATE‐UNet exhibited a slightly higher D_max_ for the body (111.2% vs. 109.8%, *p* = 0.002), although both remained well within the clinical limit of < 115%.

The sparing of OARs showed varying degrees of similarity depending on their proximity to the target volume: For the stomach and duodenum, MATE‐UNet plans resulted in slightly higher metrics compared to the reference plans. Specifically, the stomach V_39Gy_ (0.46 mL vs. 0.27 mL, *p* = 0.009) and duodenum V_39Gy_ (0.16 mL vs. 0.09 mL, *p* = 0.038) showed statistically significant differences. However, the absolute volumes for these metrics in the MATE‐UNet plans remained substantially below the clinical constraints of 1.0 mL. For OARs located further from the primary target, MATE‐UNet demonstrated performance nearly identical to the clinical reference. No significant differences were found for the spinal cord (D_max_, *p* = 0.953), kidneys (V_20Gy_, *p* = 1.000), or liver (D_mean_, *p* = 1.000).

**FIGURE 4:**
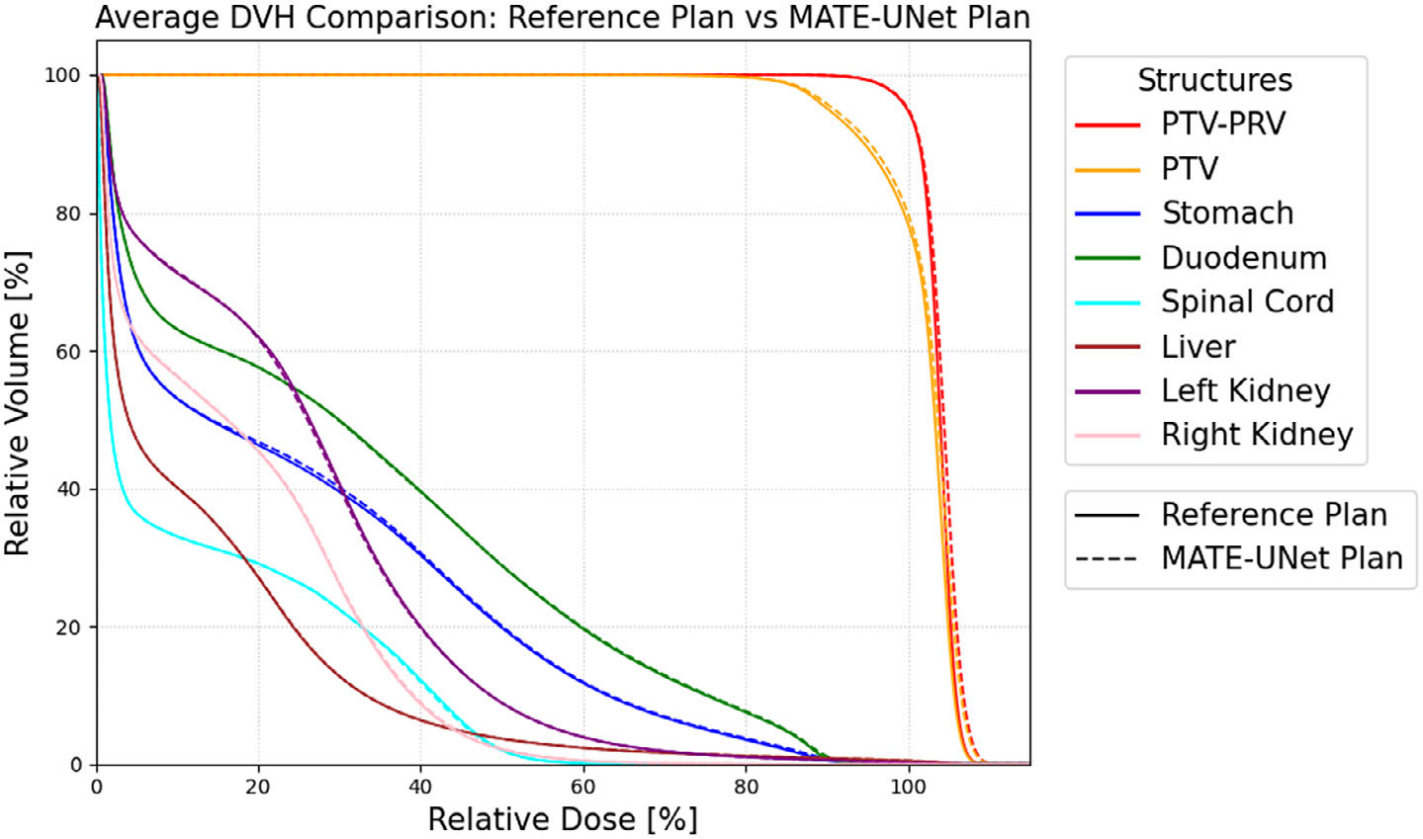
Average DVH comparison in the testing dataset between the reference plan (solid) and MATE‐UNet plan (dash).

Figure 4 illustrates the average DVH comparison between the reference plans and MATE‐UNet plans in the testing dataset. A high level of dosimetric agreement is observed across most anatomical structures, with the predicted DVH curves closely overlapping those of the clinical ground truth. Minor discrepancies are primarily confined to the target volumes (PTV and PTV‐PRV) and the stomach.

Beyond DVH‐based metrics, overall plan quality was further evaluated using the CI, HI, and GI, as summarized in Table 4. The reference plans demonstrated slightly superior performance in terms of dose conformity and homogeneity. Specifically, they achieved a significantly higher CI (0.787 vs. 0.773, *p* < 0.001) and a lower HI (0.091 vs. 0.100, *p* < 0.001) compared with MATE‐UNet. Conversely, MATE‐UNet yielded a significantly lower GI (3.73 vs. 3.80, *p* < 0.001), indicating a steeper dose fall‐off and suggesting improved sparing surrounding normal tissues at intermediate dose levels.

**TABLE 4:** CI, HI, and GI comparisons on testing dataset (20 plans) for MATE‐UNet plans and reference plans.

	Mean (SD)			
	MATE‐UNet plan	Reference plan	Pred—Ref	*p*‐value
CI	0.773 (0.032)	0.787 (0.036)	−0.013	**<0.001**
HI	0.100 (0.010)	0.091 (0.008)	0.009	**<0.001**
GI	3.732 (0.277)	3.789 (0.289)	−0.057	**<0.001**

*Note*: Statistically significant differences are marked by bold numbers in the table.

Abbreviations: CI = conformity index; GI = gradient index; HI = homogeneity index; Pred = predicted plan; Ref = reference plan; SD = standard deviation.

**FIGURE 5:**
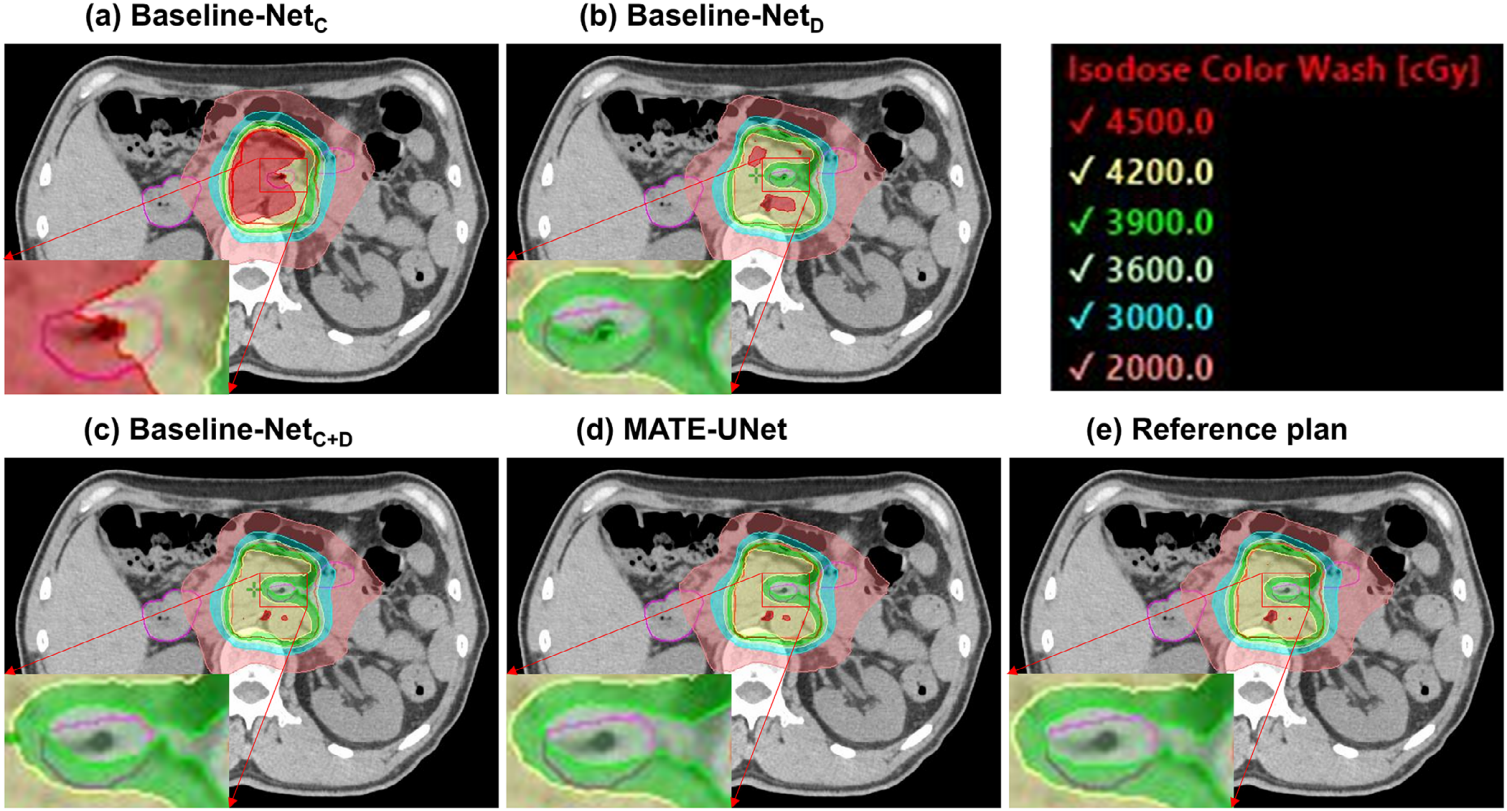
Comparison of isodose distribution for a representative testing case from the predicted plans by (a) Baseline‐NetC, (b) Baseline‐NetD, (c) Baseline‐NetC+D, (d) MATE‐UNet, and (e) reference plan. The PTV is contoured in red; the duodenum is contoured in pink.

Figure 5 provides a visual comparison of the isodose distributions for a representative testing case, consistent with quantitative analysis. Visually, the MATE‐UNet predicted plan showed a dose distribution comparable to that of the reference plan. It exhibited both superior sparing of the duodenum (contoured in pink) and a visibly tighter 36 Gy isodose line (green area) compared to Baseline‐Net_C+D_, as indicated in the magnified inserts. In contrast, the other baselines exhibited poorer conformity, characterized by expanded 45 Gy hot spots (red area) and extensive high‐dose spillage.

**FIGURE 6:**
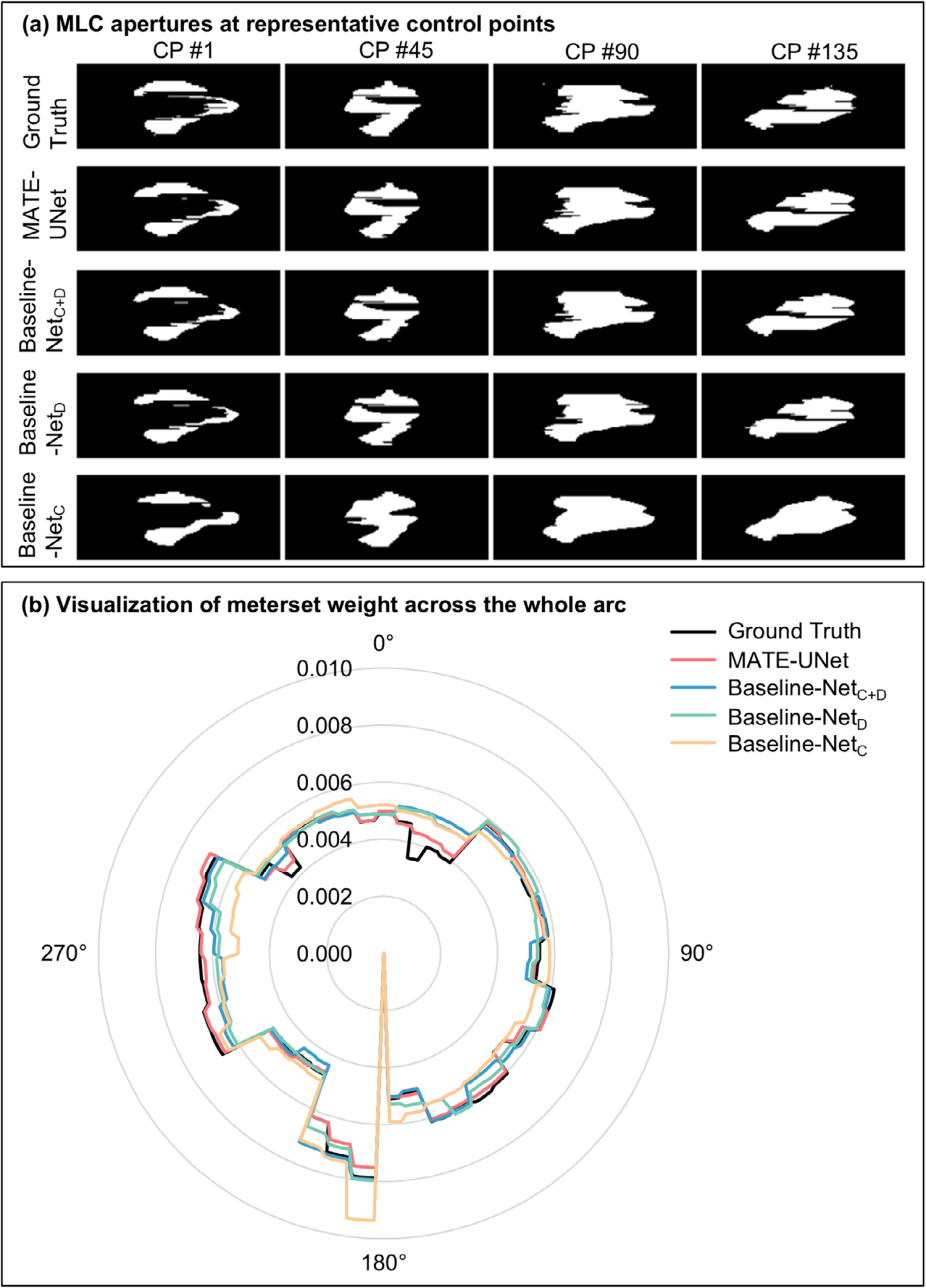
Comparison of predicted MLC apertures and meterset weights. (a) Visual comparison of MLC apertures at representative control points (CP #1, #45, #90, #135) among Ground Truth, MATE‐UNet, and baseline models. (b) Visualization of meterset weight distribution across the whole arc, comparing the proposed MATE‐UNet against Ground Truth and baseline models.

Additionally, a visual comparison of MLC apertures and meterset weights among the proposed model, baselines, and ground truth is provided in Figure 6.

Finally, in terms of computational efficiency, the average times required for data pre‐processing and for the proposed MATE‐UNet to generate a DICOM plan file (including inference and post‐processing) were 88.5 ± 0.5 and 4.2 ± 0.1 seconds per patient, respectively.

## 4 DISCUSSION

In this study, we demonstrated the efficacy of MATE‐UNet, an optimization‐free framework for predicting VMAT parameters in complex pancreatic cancer cases. By directly mapping multi‐modal BEV projections (dose and contours) to MLC apertures and MU sequences, the model effectively bypasses the traditional inverse optimization bottleneck while maintaining clinical acceptability.

### 4.1 Clinical acceptability and comparative dosimetric analysis

The clinical viability of MATE‐UNet is primarily demonstrated by its 100% clinical goal success rate, indicating that the framework can consistently produce plans that satisfy stringent radiotherapy requirements. However, a comprehensive DVH comparison reveals that MATE‐UNet plans generally exhibit an upward dose trend relative to the reference plans. The statistically significant differences observed in the body D_max_, stomach V_39Gy_ and duodenum V_39Gy_ suggest that the current model remains slightly inferior to expert manual optimization when managing highly complex anatomical structures. This observation is further supported by the superior CI and HI achieved by the reference plans, which indicate a higher prevalence of localized hot spots in the MATE‐UNet dose distributions. This discrepancy likely stems from the fundamental difference in the planning process: unlike traditional inverse optimization, which involves an iterative “trial‐and‐error” weight‐tuning to refine dose boundaries, our framework relies on a direct, one‐step inference. Consequently, minor inaccuracies in the prediction of fine MLC apertures may manifest as increased dose heterogeneity within the target volume. Despite these localized hot spots, MATE‐UNet demonstrated a statistically superior GI compared to the reference plans. This steeper dose fall‐off effectively compensates for the internal hot spots by ensuring more rapid dose reduction in the transition zone outside the target. This unique physical characteristic explains why MATE‐UNet remains capable of satisfying all clinical constraints even in cases where the internal homogeneity is less optimal than the clinical benchmark. These findings suggest that while fine‐scale leaf positioning requires further refinement, MATE‐UNet already functions as a robust engine for high‐quality LINAC parameter generation.

### 4.2 Multi‐modal BEV input and spatial‐temporal architecture

The use of BEV projections establishes a direct geometric mapping between the network input and the LINAC parameters of VMA. Unlike volumetric 3D inputs, BEV images align with the 2D modulation space of MLC leaves, bypassing the inefficient dimensionality reduction typically required to derive apertures. Our ablation study clarifies the distinct roles of these modalities: While dose projections provide the fundamental guidance for intensity modulation, anatomical contours supply the requisite spatial constraints to limit high‐dose spillage. The integration of both modalities ensures that the model can simultaneously achieve target coverage and satisfy stringent proximity constraints, particularly in the critical intersection between the PTV and the gastrointestinal PRVs. This finding is consistent with previous study conducted by Vandewinckele et al.,^39^ which demonstrated that combining anatomical representations with dose information yields optimal predictive performance. The architectural design of MATE‐UNet addresses the distinct requirements of MLC and MU prediction through a multi‐task framework. For MLC aperture prediction, the Feature Fusion and Attention Gate modules facilitate the extraction of fine‐scale edge features. This architectural refinement is particularly evident in the improved conformity and refined aperture definitions at target‐OARs boundaries. More importantly, the integration of a Transformer module addresses the inherent temporal nature of VMAT delivery. Although positioned at the network's bottleneck, the Transformer processes compressed feature maps derived from the input BEV projection sequence, which naturally retains its spatiotemporal properties. Unlike standard convolutional networks that focus on local features, this architectural design enables the model to capture global spatiotemporal correlations across the entire arc. Such a comprehensive understanding of the delivery sequence greatly improves the overall accuracy of the generated LINAC parameters, particularly in predicting the MU matrix, thereby contributing to the superior OAR sparing and high clinical acceptability observed in this study.

Placed within the broader context (Table 5), our results validate the specific design choices essential for an optimization‐free workflow. First, MATE‐UNet distinguishes itself from standard DL approaches that predict intermediate dose^14^ or fluence^16^ by directly predicting the machine parameters. Second, comparisons with prior BEV‐based studies support the necessity of the proposed multi‐modal and architectural strategy. While Ni et al.^18^ and Vandewinckele et al.^19^ explored direct prediction, they relied primarily on anatomical contours and required post‐optimization to fulfill the clinical requirements.

**TABLE 5:** Comparison of previous related studies.

Study	Clinical protocol	Input	Output	Model	TPS optimization	Clinical performance
Fan et al^14^, 2019	Head and Neck IMRT (9 beams, 51.0‐70.4 Gy)	3D CT and contours	3D dose distribution	ResNet‐antiResNet	Required	Comparable to manual plans
Arberet et al, ^16^ 2025	Prostate VMAT (single arc, 66.5 Gy)	BEV projections of dose	Fluence map sequence	3D MedNeXt with residual ConvNeXt	Required	High dosimetric agreement with clinical plans
Ni et al,^18^ 2022	Prostate VMAT (single arc, 60.0 Gy)	BEV binary masks of contours	MLC apertures	3D U‐Net	Required	Statistically equivalent to reference plans
Vandewinckele et al,^19^ 2023	Breast VMAT (3 arcs, 45.57 Gy)	BEV projections of contours	MLC apertures and MU	cGAN (3D U‐Net + PatchGAN)	Required	Direct prediction failed to meet clinical requirements
Heilemann et al,^20^ 2023	Prostate VMAT (single arc, 60.0‐78.0 Gy)	3D dose distribution	MLC/Jaw positions and MU	3D ResNet‐based CNNs	Optimization‐free	High delivery accuracy (Gamma pass rate ∼92%), but inferior target coverage
Heilemann at al,^21^ 2025	Prostate VMAT (single arc, 60 Gy)	3D CT and structure arrays	MLC/Jaw positions and MU	3D U‐Net + 3D ResNet‐based CNNs	Optimization‐free	Almost meet all clinical requirements (except V_60Gy_ and dose heterogeneity)
This work	Pancreas VMAT (single arc, 42.0 Gy)	BEV projections of dose and contours	MLC apertures and MU	MATE‐UNet	Optimization‐free	100% clinically acceptable

Abbreviations: BEV = beam's eye view; IMRT = intensity modulated radiation therapy; MLC = multi‐leaf collimator; MU = monitor unit; VMAT = volumetric modulated arc therapy.

Furthermore, the advantage of the BEV representation becomes evident when compared with 3D‐based approaches. Heilemann et al.^20^ attempted direct parameter prediction from 3D volumetric inputs but reported inferior target coverage relative to clinical plans. Although their more recent work successfully established an end‐to‐end auto‐planning framework, challenges remained in terms of dose heterogeneity and high‐dose sparing according to multi clinical cretiria.^21^ Regarding computational efficiency, the average inference time of the proposed framework is approximately 4.2 s. In existing literature,^14^, ^16^, ^19^, ^20^ reported inference times for DL‐based prediction vary considerably, ranging from 19 ms to approximately 10 s depending on model complexity and hardware configurations. When fully accounting for the data pre‐processing phase, the proposed method requires a total of approximately 93 seconds per patient. Compared with semi‐automated planning systems, which typically require tens of minutes for plan generation,^40^, ^41^ the proposed method still offers a substantial reduction in overall computation time. However, it is objectively acknowledged that our 93 seconds total runtime is slower than the latest fully end‐to‐end frameworks; for instance, Heilemann et al.^21^ recently reported a complete end‐to‐end workflow with a runtime of approximately 38 s. Unlike this end‐to‐end system, our current study positions MATE‐UNet as a downstream component within a fully automated planning pipeline. To realize a complete “anatomy‐to‐plan” framework, our method is designed to be integrated with an independent upstream dose prediction model.

Despite these encouraging findings, we acknowledge several limitations in the current study. First, the dataset utilized in this study was standardized to a specific clinical protocol: a prescription of 42 Gy in 15 fractions, delivered via a single full arc (180 control points) with a fixed 30° collimator angle. While this controlled setting facilitated the initial validation of the methodology, we acknowledge that clinical protocols vary, often involving dose escalation strategies or different fractionation schemes. Future work will therefore expand the training cohort to include a broader range of prescriptions and beam configurations, ensuring the model's generalizability across diverse treatment scenarios. Second, the model predicts CMWs as relative values rather than absolute MUs. Consequently, an additional normalization step is required to determine the absolute MU scale before a clinically usable final treatment plan can be generated. Therefore, the current framework should be regarded as a valuable step toward bridging, rather than as having fully bridged, the gap between the desired dose distribution and the final treatment plan. This requirement is consistent with prior literature; for example, although Heilemann et al.^20^ directly predicted absolute MU values, their generated plans still required a corrective normalization to accurately match expected clinical doses. Importantly, the deterministic rescaling required in our framework is distinct from iterative optimization, typically takes less than one minute, and therefore has a limited impact on workflow efficiency. This study primarily focused on dosimetric accuracy. Experimental QA metrics, such as Gamma pass rates from phantom‐based measurements, were not reported. While the current results provide a dosimetric proof‐of‐concept, future work will involve evaluating delivery accuracy within a comprehensive “anatomy‐to‐plan” system to ensure clinical reliability. Finally, clinical dose distributions were used as inputs to validate the performance of MATE‐UNet independently. To realize a fully automated “anatomy‐to‐plan” pipeline, future work will integrate this framework with an upstream dose prediction model, replacing clinical reference doses with predicted dose distribution.

## 5 CONCLUSIONS

This study demonstrates the feasibility of MATE‐UNet for the direct prediction of relative VMAT parameters without iterative optimization in complex pancreatic cancer cases. By leveraging multi‐modal BEV projections and a Transformer‐enhanced architecture, the proposed framework represents a valuable step toward bridging the gap between the desired dose distribution and a clinically usable treatment plan, while reducing reliance on iterative optimization. Our findings indicate that integrating anatomical and dosimetric information is essential for accurate VMAT parameter prediction. Although an additional step is required to determine the absolute MUs from the predicted relative values, MATE‐UNet shows potential to serve as a downstream component of a future fully automated anatomy‐to‐plan pipeline, laying the foundation for more efficient and consistent clinical workflows.

## CONFLICT OF INTEREST STATEMENT

The authors declare no conflicts of interest.
